# Implementation of a quality and safety checklist for haemodialysis sessions

**DOI:** 10.1093/ckj/sfu145

**Published:** 2015-01-12

**Authors:** Daniele Marcelli, Antero Matos, Francisco Sousa, Ricardo Peralta, João Fazendeiro, Angel Porra, Victor Moscardo, Maria Teresa Parisotto, Andrea Stopper, Bernard Canaud

**Affiliations:** 1 EMEALA Medical Board, Fresenius Medical Care, Bad Homburg, Germany; 2 NephroCare, Lisbon, Portugal; 3 NephroCare Coordination, Fresenius Medical Care, Bad Homburg, Germany

**Keywords:** checklist, haemodialysis, patient safety, quality improvement, quality tool

## Abstract

**Background:**

Patient survival and quality of life depend on each haemodialysis session being performed without fault. Monthly assessments of dialysis dose adequacy often fall short of this. This study reports the results of a feasibility study for the achievement of improved safety and quality in a haemodialysis session with the implementation of a 15-point checklist.

**Methods:**

Fifteen quality indicators were compiled and tested in a Portuguese dialysis clinic from 1 February 2012 to 30 June 2013. The checklist was completed by the nursing staff and comprised three parts: Pre-session Safety Checks; Session Initiation Checks and Post-session Quality Checks. The maximum score that could be reached per session was 15.

**Results:**

One hundred and twenty-eight patients were distributed over 2–3 shifts. Of the 16 nurses employed, 4 were full time. The final average score was between 14 and 15. No nurse-specific and no shift-specific significant differences were detected. Four issues were identified that had a major effect on the results as a whole: delays in connection time; incompletely delivered treatment time; non-achievement of final body weight and failure to reach a *Kt/V* of at least 1.4. Improvements were most consistent in the Monday–Wednesday–Friday morning shifts compared with other shifts, and were temporarily compromised by the opening of a new shift.

**Conclusions:**

The implementation of checklists for haemodialysis is feasible in routine clinical practice, even in clinics where only part of the staff is employed full time. The application of such checklists enhances the overall quality and safety of the delivered treatment.

## Introduction

Patient safety and quality of life are sensitive to each dialysis session being performed without fault. In current performance-rewarded dialysis provision systems (e.g. the Portuguese and German healthcare systems), pay-for-performance (P4P) measures for dialysis quality are based on monthly assessments of performance indicators, such as dialysis dose adequacy and adherence to prescribed treatment time [1–3]. Such monthly assessments fall short of assuring highest dialysis quality for every single session. For example, prescribed treatment time can be very different from the actual treatment time in a single session, as patients can be disconnected prematurely for several reasons, ranging from hypotension to the simple request of the patient. However, it should be pointed out that such safety and quality deviations are rarely due to the lack of professional knowledge, but are rather the result of poor organization, verification, coordination, communication and a general lack of a common culture of safety and quality [4].

Unfortunately, haemodialysis provision is generally considered to be standard treatment that is delivered at a prescribed time and interval to deliver the targeted dose. Rarely are the full nursing professional skills employed.

However, quality is not only the outcome, especially from the patient perspective. Time spent waiting for connection is rarely enjoyed by patients [5], especially when there is uncertainty about the duration. Patients in the waiting room should know how long they have to wait, and this should not exceed a certain reasonable amount of time. Furthermore, also from the patient's point of view, quality should also encompass the absence of treatment-related complications. Treatment tolerance has been addressed in some studies [6, 7]. However, considering how frequently patients are treated, it is deserving of a weighting similar to that of survival. Unfortunately, in reality, it is considered to be quite normal that patients have unpleasant experiences during treatment, such as cramps, hypotension and bleeding. Consequently, it can even be difficult to get nurses to report these complications since they are considered to be normal.

These issues are not new in medicine or in other clinical processes, e.g. in elective surgery. The WHO has already raised attention to the need to improve safety by using checklists [8]. Accordingly, checklists were implemented in eight hospitals located in Canada, India, Jordan, New Zealand, the Philippines, Tanzania and the UK, resulting in a concomitant reduction in the rate of death and complications [9]. More recently, French healthcare authorities have promoted the use of checklists in their recommendations of good practice in different medico-technical activities. Galland *et al*. [10] followed this recommendation by applying a checklist in the dialysis room focusing on the safety aspect of the haemodialysis session, in particular by optimizing and regulating the communication between nurses and physicians. Following the suggestion of the WHO, and unaware of the Galland initiative, we prepared a checklist considering safety and quality aspects of the extracorporeal dialysis session.

Here, results of a feasibility study for the application of this system in the clinical setting are reported, defining as success the completion of each dialysis session with all targets reached.

## Materials and methods

Fifteen quality indicators were defined as the result of intensive nurse–nephrologist discussions with the focus on aspects relevant to safety and quality in a dialysis session. The checklist was comprised three parts: (i) pre-session safety, (ii) session initiation and (iii) post-session quality. The maximum score obtainable per session was 15, and nurses were informed of the result achieved immediately at the end of the session. The checklist is summarized in Table 1.

**Table 1.** Checklists for safety and quality in dialysis treatment

Extracorporeal dialysis checklist
A. Pre-session Safety Checks
1. Review of possible complications in the interdialytic period
2. Patient vascular access arm correctly washed?
3. Vascular access assessment for patients with arteriovenous fistula or graft
4. Search for signs of infection in case of catheter
5. Absence of residual disinfection agent
B. Session Initiation Checks
1. Initiation of treatment within 15 min from the scheduled time
2. Check that needle size is as prescribed
3. Check that dialyser type is as prescribed
4. Check that all dialysis parameters are entered as prescribed
5. Check that the patient does not receive unnecessary punctures.
C. Post-session Quality Checks
1. Check that blood pressure and heart rate measurements were recorded as planned
2. Check for the absence of complications related to possible errors (dialyzer coagulation, blood loss and needle dislodgement)
3. Check that the prescribed treatment time was delivered with a maximum tolerance of 10 min.
4. Check that the prescribed dry body weight was reached^a^
5. Check that the minimal dialysis dose was reached, i.e. *Kt/V* from ionic dialysance or total reinfusion in case of on-line HDF reached

^a^Defined as the difference between the achieved, final body weight and the dry weight of <1.0 kg (sessions 1 and 2 of the week) and < 0.5 kg (session 3 of the week).

Some of the required information (e.g. time of start of treatment, blood pressure, *Kt/V* and post-dialysis body weight) was automatically acquired by integrated dialysis equipment systems (i.e. weighing scales and dialysis machine) using the European Clinical Database (EuCliD) management software [11]. Dialysis dose (*Kt/V*) was calculated by the Online Clearance Monitor (OCM) based on ionic dialysance [12].

The checklist system was implemented as a traffic light system, and applied for execution after each single dialysis session recorded into the EuCliD database. At the end of the session, a green light signalled the full achievement of all checkpoint items for a session to the responsible nurse. A yellow light indicated failure to achieve one target, and the red light showed that more than one target was missed. The data were stored in the EuCliD database and were then also available for the monthly reporting of results.

For this study, data were extracted in anonymous form from the EuCliD database where data are collected according to company standard clinical protocols and procedures [11]. All patients consented in writing to the use of this data for scientific research.

### Statistical analysis

Normally distributed continuous variables are described using means and standard deviations; median and 25th–75th percentiles are used otherwise. Categorical variables are described as percentages.

## Results

One hundred and twenty-eight patients were treated from 1 February 2012 to 30 June 2013 in the participating Portuguese clinic. Patient characteristics are reported in Table 2. Patients were initially distributed over two shifts on Mondays, Wednesdays and Fridays and over three shifts on Tuesdays, Thursdays and Saturdays. In November 2012, a third shift was added on Mondays, Wednesdays and Fridays. Sixteen nurses were employed in the clinic, four full time and the others part time. On average, nurses worked in this clinic 9 days a month. The patient-to-nurse ratio was between 4 and 5.

**Table 2.** Baseline characteristics of the patients treated in the clinic from 1 February 2012 to 30 June 2013

Variable	
Age (years; mean, SD)	69.2 ± 15.4
Gender (female; %)	46.7
Cause of renal disease	
Glomerulonephritis (%)	10.8
Diabetic nephropathy (%)	12.5
Cystic kidney disease (%)	2.5
Chronic pyelonephritis (%)	5.0
Vascular disease (%)	31.7
Miscellaneous (%)	8.3
Unknown (%)	29.2
Diabetes (present; %)	20.8
Comorbidities	
Coronary artery disease (%)	3.3
Congestive heart failure (%)	12.5
Peripheral vascular disease (%)	8.3
Cerebrovascular disease (%)	14.2
Chronic pulmonary disease (%)	4.2
Tumour (%)	13.3
Charlson comorbidity index (mean ± SD)	3.2 ± 1.3
Age-adjusted Charlson comorbidity index (mean ± SD)	5.7 ± 2.3
Dialysis vintage (months); (median, 25th–75th percentile)	18; 3–67
Vascular access	
AV fistula (%)	61.3
Catheter (%)	27.7
Graft (%)	10.9

**Fig. 1.**
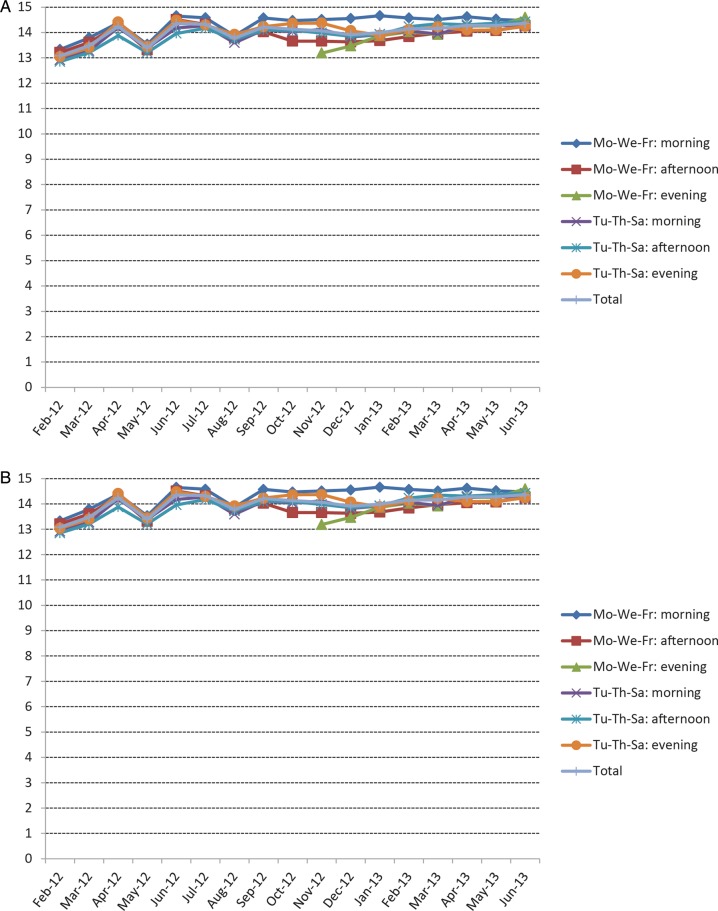
Average monthly score by participating nurse (**A**) and by shift (**B**).

**Fig. 2.**
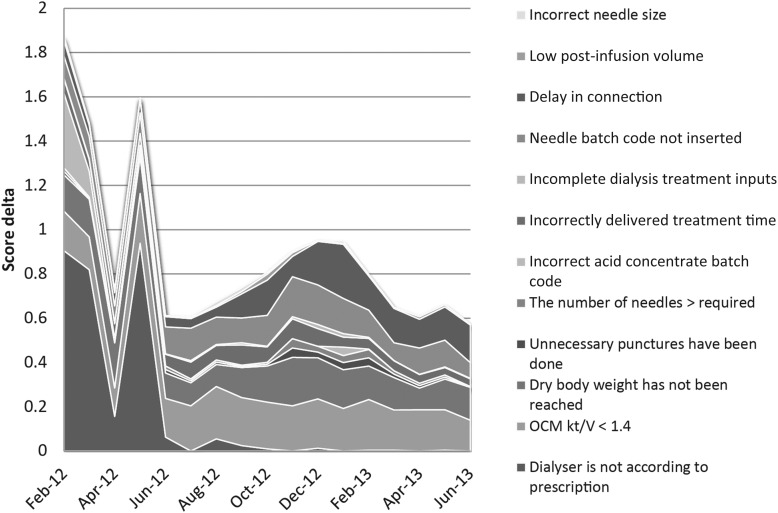
Score delta, defined as the maximum score (i.e. 15) minus the achieved score, by month and by issue.

**Fig. 3.**
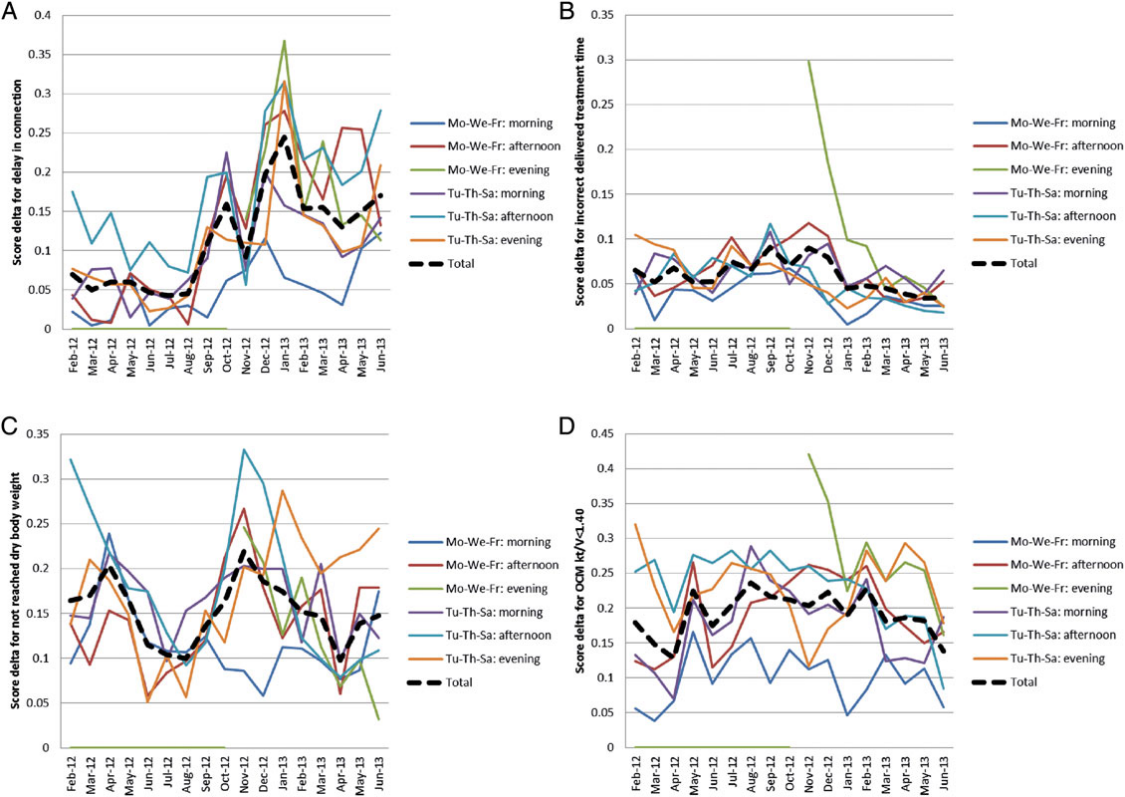
Score delta, defined as the maximum score (i.e. 15) minus the achieved score, due to (**A**) connection after >15 min from the scheduled time by shift, (**B**) incompletely delivered dialysis treatment time by shift, (**C**) differences between prescribed and achieved final body weight by shift and (**D**) OCM *Kt/V* lower than 1.40 by shift.

As reported in Figure 1A, the average monthly score rose from the initial 13 to the final result that was between 14 and 15. No significant nurse-specific differences were detected. Figure 1B shows the development of the monthly average score by shift. At the end of the pilot study, no significant shift-specific differences were detected, but it should be underlined that at the opening of the new third shift in November 2012, the average score was in the same range as the score of the other shifts at the start of the study. Figure 2 shows the reasons for the lower score. The initial phase, which was highly variable, was mainly influenced by inconsistencies between the documented and the applied dialyser. These were largely resolved during the course of the study, while similar technical issues regarding needle batch code inconsistencies persisted. Figure 3A shows the details of the major issues affecting results by the shift. Delays in connection time were under control up to August 2012 and then deteriorated, mainly in the afternoon and evening shifts. Incompletely delivered treatment time was under control throughout the full pilot study, moving from an initial failure rate of 6.5% to the final 3.5%. The opening of the new third evening shift was associated with a temporary deterioration of the results (Figure 3B). The failure rate associated with non-achievement of the final body weight decreased from 16.4 to 14.8% between February 2012 and June 2013. By April 2012, this failure rate had already decreased to 9.8%, but peaked again in November 2012 to 21.9% (Figure 3C). On average, the failure rate due to *Kt/V* being below 1.40 decreased from 17.9 to 13.8% in the course of the study period. A Monday–Wednesday–Friday morning shift was always associated with the best performance (Figure 3D).

## Discussion

This pilot study on the implementation of a checklist to ensure high safety and quality in each dialysis session showed that renal nurses are motivated to engage, and that this is feasible even for nurses who work only 4–5 days a month. Patient characteristics were representative of a western European dialysis population.

The final average checklist score was between 14 and 15, close to the complete achievement of all of the checkpoints. It was similar among the participating nurses, with a positive trend over time. No significant differences were detected between the different shifts.

Initially, some critical problems appeared impossible to solve. The most critical was the patient waiting time before connection, which was targeted to be limited to a maximum of 15 min delay from the scheduled time. In this clinic, located in a suburb of Lisbon, patients came to the clinic by common transportation in groups of 5–6. Sometimes, a number of minibuses arrived together and patients expected the nursing staff to connect them as soon as possible. This process was completely reorganized. Patients received a connection time schedule for each of the weekly sessions and the minibus company was advised to bring patients at a given time in order to balance the waiting time over the week and to avoid exceeding the limit of 15 min. Patients accepted this change and, subsequently, the normally stressful connection time became more relaxed. This change also allowed nurses more time to perform the pre-session safety checks they are expected to do. It was observed that, after the initial enthusiasm associated with the involvement in the pilot study, later-than-planned connections increased to a final 142% higher failure rate. This was possibly due to the pressure associated with the opening of the new evening shift and the engagement of new nurses. This pilot study demonstrated that it is possible to find solutions to complex problems with the involvement of the professionals who are perfectly aware of the multifaceted dimensions of the issue at hand. A further lesson of this pilot study is that the process naturally tends to return to the traditional less organized approach. Therefore, to maintain the results for the long term, there is a need to revise the situation periodically, probably every 1–2 months, under the light of new patients and changes in shift allocation. One should also consider that many components of the dialysis process appear fixed by tradition. Only when the staff is challenged by the results of such a checklist, does it become possible to discuss and implement process improvements.

Many statistical studies have shown an association between the prescribed treatment time and outcome, but it is fundamentally the good intention of the medical staff that is actually correlated with the delivered treatment time. Adherence to prescribed treatment time was a priority in the tested Portuguese clinic, and the proportion of sessions with a deviation of delivered treatment time greater than 10 min decreased to 3.5%. This was proven with the automatic transfer of information from the dialysis machine to the computer, avoiding any possible manipulation.

The dry body weight target, with the maximum deviation of 0.5 kg at the third session of the week, was finally achieved in >85% of the sessions. Interestingly, in summertime the results improved up to 90% (Figure 3C). It should be underlined that the correct achievement of dry body weight target is not only associated with a better outcome but also with a reduction in costs due to the association with avoidable emergency dialysis overnight or during weekends [13].

Dialysis dose has long been considered the main indicator of quality of treatment [14]. Accordingly, major significance has been given to it by different healthcare organizations [1–3]. However, it is normally measured once a month, so the evaluated session can potentially be different from the other 12–13 sessions. In this project, dialysis dose in all sessions was evaluated by the OCM device, which measures urea clearance during treatment via sodium dialysance [12]. According to this, which is again automatically transferred from the machine to computer, the target of single pool *Kt/V* ≥1.40 was reached in over 86% of the sessions, a result in line with the requirements of the good practice guidelines. The main reason for failure was the presence of a catheter as vascular access, a limitation already discussed in many papers [15].

Other reasons for failing to achieve all 15 points in the checklist were of a technical nature, i.e. erroneous algorithms or code mismatches of prescribed disposables, i.e. dialysers, particularly affecting the results at the beginning of the pilot study. After correction of these technical problems involving the proper maintenance of the code in the IT system, the residual mismatches between the prescribed disposables and those actually used were reduced significantly, i.e. the proportion of incorrect needle size was reduced to the range of 1 per 1000. Other important issues, specifically those related to the pre-session phase, were solved automatically during the pilot study simply through reminding the nurse of certain tasks to be performed at each of the three steps.

As previously mentioned, our experience with the use of a checklist for safety and quality aspects in dialysis is not unique. A similar approach was tested by another group in France [10]. Interestingly, the two separate studies in France and Portugal produced very similar results. In the pre-session stage, several checks in both studies are related to safety aspects focusing on the verification of the proper setting of the equipment and on the problems reported by the patient. Regarding the Session Initiation phase, both checklists focus on treatment time. In our tool, we target a maximum deviation of 10 min from the target, while the French study awards different numbers of points for treatment time deviations of 5–15 and >15 min. Other features common to both checklists were the avoidance of unnecessary AV-fistula punctures, the achievement of at least minimal *Kt/V* and attainment of the prescribed dry body weight. In total, 70–80% of the items included in our tool were somehow considered also in the French checklist. Notably, the French study also adopted the ionic clearance method for monitoring of *Kt/V* in all sessions. This methodology avoids pre- and post-dialysis blood sampling, so it can be applied in all of the sessions. The main difference between the two approaches was the importance allocated to the patient experience in our checklist, i.e. the inclusion of containment of patient waiting time. Galland *et al*. [10], on the other hand, assigned more relevance to nurse–physician communication. It should be mentioned that checklists have already previously been proven to be a valid tool in improving communication between nurses, surgeons and anaesthesiologists [16].

In conclusion, this pilot study demonstrated that it is possible to implement a checklist that improves safety and quality of the dialysis treatment, even in clinics with many part-time nurses.

## Conflict of interest statement

All authors are employees of Fresenius Medical Care and may hold stock in the company.

(See related article by Bray and Metcalfe. Improving patient safety in haemodialysis. *Clin Kidney J* (2015) 8: 262–264.)
